# Comparison of the simplest diets to find the most effective one in developing ant colonies of *Lasius niger*

**DOI:** 10.1038/s41598-025-06406-x

**Published:** 2025-07-01

**Authors:** Norbert Szabó, Jenő Nagy, András Tartally

**Affiliations:** 1 Department of Evolutionary Zoology and Human Biology, University of Debrecen, Egyetem tér 1, Debrecen, H-4032 Hungary; 2 Juhász-Nagy Pál Doctoral School of Biology and Environmental Sciences, University of Debrecen, Egyetem tér 1, Debrecen, H-4032 Hungary; 3 HUN-REN-UD Conservation Biology Research Group, Department of Botany, University of Debrecen, Egyetem tér 1, Debrecen, H-4032 Hungary

**Keywords:** Cockroach, Cricket, Black garden ant, Hibernation, Honey, Mealworm, Sucrose, Wintering, Biological techniques, Developmental biology, Entomology

## Abstract

Nurturing, especially feeding ant colonies is a vital topic among ant researchers and ant keepers. Numerous factors contribute to growing healthy ant colonies rapidly, including, for instance, humidity levels and appropriate farming methods. Proper diet is also an essential consideration. Many articles contemplate the potential of artificial diets. Variable carbohydrate-based diets have been tested but the role of protein has largely been overlooked. We aimed to analyse the effects of these diets to be able to standardize and compare results of different laboratories. We assessed the most readily available, natural, inexpensive, known, and efficient diets to ensure optimal nutrition for ant colonies by analysing the development of 100 *Lasius niger* colonies. They were given four different carbohydrate-based diets in the first year and four different protein-based diets in the second year. Based on our findings, honey water was the best carbohydrate source. The best protein diet, however, consisted of crickets, which positively influenced not only the development but also the survival success of the colonies during the wintering period. Thus, we suggest rearing laboratory colonies of *L. niger* on honey water and crickets. It would be worth repeating this study with different ant species and doing biochemical analysis on the diets.

**Supplementary Information:**

The online version contains supplementary material available at 10.1038/s41598-025-06406-x.

## Introduction

Ants (Hymenoptera: Formicidae) have a large biomass. They constitute an estimated 15–20% of global fauna^1^. They therefore play important roles in natural and agricultural terrestrial communities, and their eusocial life form is the focus of interest of many scientific topics^2^. The ability to effectively and efficiently rear ant colonies is essential to research in many areas of myrmecology. It is important therefore to know whether differences in ant colony diets have an effect on the productivity and survival success of ant colonies grown in laboratories.

For most ant species, the main sources of carbohydrates are plant exudates, nectar, and honeydew^3–6^. As e.g. *Lasius niger* (Linnaeus, 1758) workers prefer tri- and disaccharides over monosaccharides^7,8^, table sugar solutions can be appropriate as a basic carbohydrate source under laboratory conditions^4,5,9^. We also know that sugar-based diets are determined by several criteria, such as digestibility, energy content, viscosity, and molarity^9–11^. Tri- and disaccharides are chosen over monosaccharides by *Lasius niger* scouts^7,8^ because di- and oligosaccharide molecules contain two to three times more energy than monosaccharides, despite the fact that digestive enzymes are needed to metabolize them^9,11^. At the same weight concentration of different sugar solutions, molarity and viscosity are higher in the case of monosaccharide solutions than di- and oligosaccharide solutions^10^and high viscosity has a negative effect on fluid intake rate^12^. Different types of honey contain different mono-, tri-, and disaccharides in different proportions^13,14^ and are often used to feed artificial ant colonies, usually in the form of honey water^8,15–18^. All in all, the most attractive sugar solutions for ants are those which provide high energy content and are easy to break down but have less viscosity, such as the approximately 1.75 mol/L (36.75% w/w) sucrose solution^19^.

In addition to sugars, proteins are also important for the ant colonies, as they are essential to the successful development of offspring and the queen’s ability to produce eggs^20–22^. Most often, proteins are provided by crickets, cockroaches, fruit flies, or mealworms. To ensure better nutrition of ant colonies grown in laboratories, some artificial diets have been created^15,16,23–25^. Artificial diets, however, have been shown to have drawbacks. When these diets do not include insect supplements, for instance, this leads to moderate colony growth and fewer offspring^26^.

**Fig. 1:**
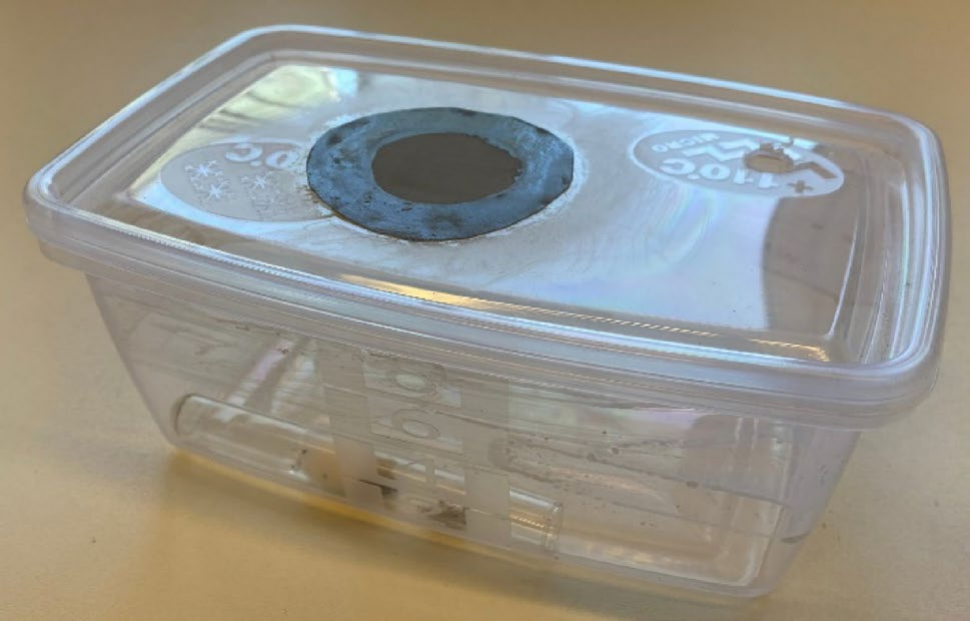
A plastic box nest in which a laboratory ant colony was kept.

Given these considerations, our aim was to compare the effectiveness of different, familiar, and easily available and producible natural diets on the development and hibernation of laboratorial ant colonies (Fig. 1). Based on 35 years of intensive ant rearing experience, we thought that sugar solutions containing honey would be the best carbohydrate source for ant colonies and the best supply of simple proteins would be cockroaches or crickets.

## Results

**Fig. 2:**
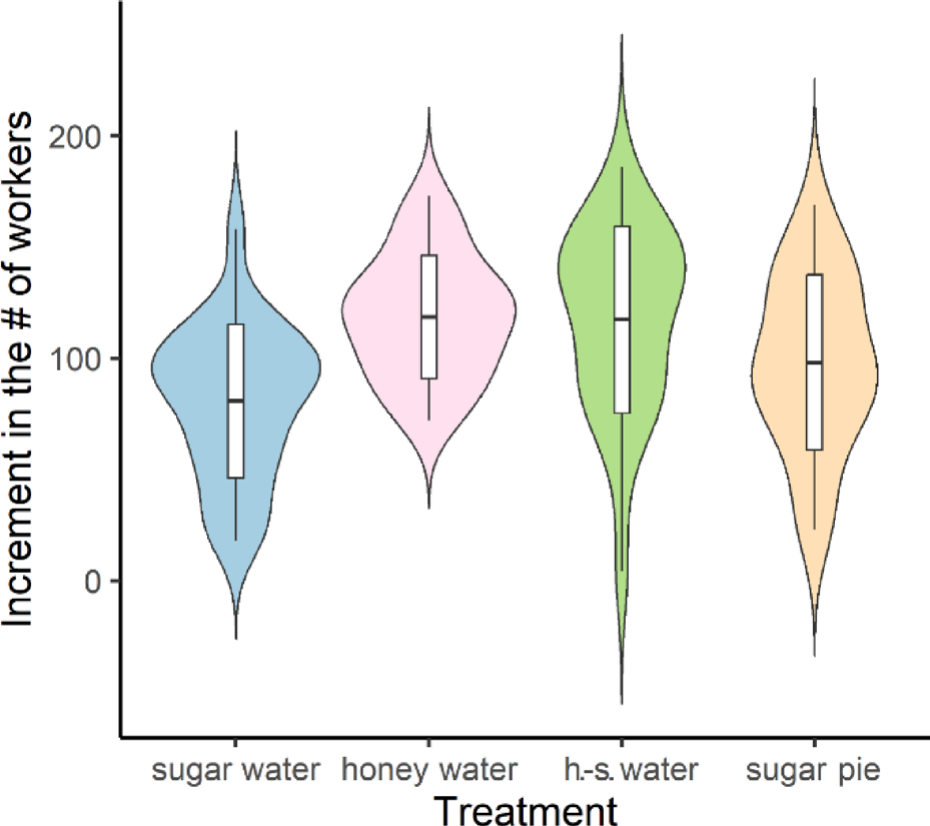
Increment in the number of workers during different carbohydrate treatments. Boxes indicate mean and standard deviation values within each group.

**Fig. 3:**
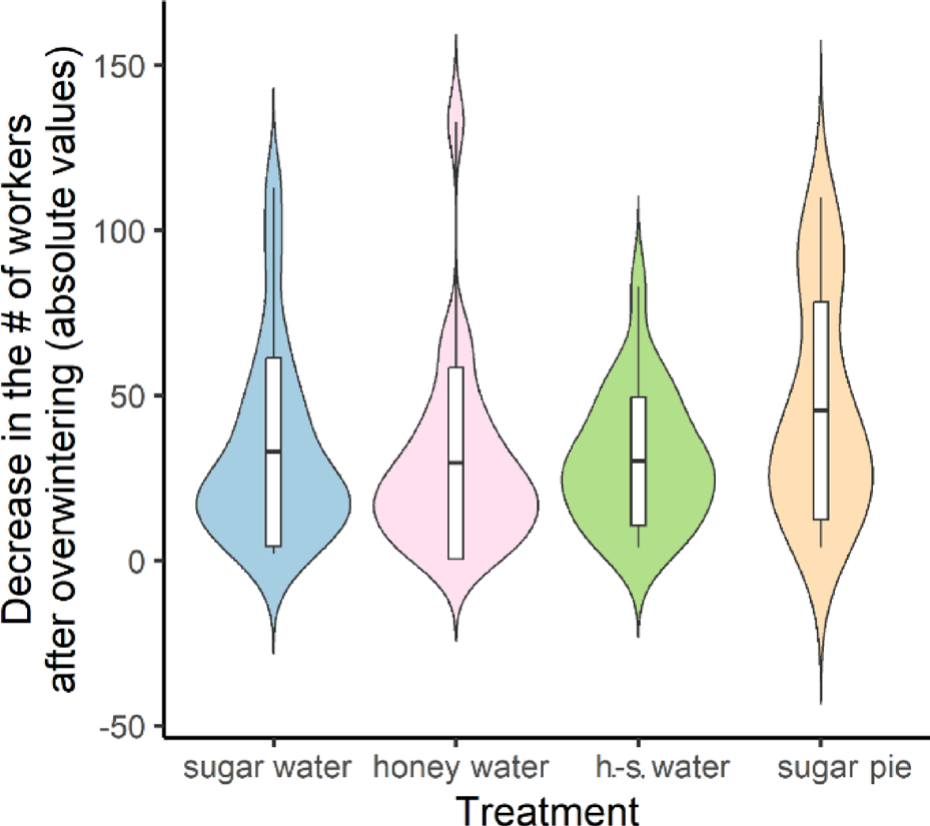
Decrease in the number of workers (absolute values) after the wintering period of colonies on carbohydrate diets. Boxes indicate mean and standard deviation values within each group.

Increases in the number of workers in *Lasius niger* colonies fed different carbohydrate diets showed normal distribution (Shapiro-Wilk’s test: W = 0.98, *p* = 0.27) with equality of variances between the groups (Levene’s test: F = 0.98, *p* = 0.41). The linear regression model indicated statistically significant differences among treatments (Table 1), which were confirmed by the *post hoc* comparisons (Fig. 2; Table 2). These results suggest that a carbohydrate diet containing honey could increase colony size better than other sources. Drops in the number of workers after the wintering period in these colonies followed a negative binomial rather than a normal distribution (W = 0.89, *p* < 0.001). According to the negative binomial regression model (Table 1) and the *post hoc* tests, the colonies had statistically similar losses (Fig. 3; Table 2).

**Table 1:** Summary results of the regression models investigating the effect of treatments on the number of workers during feeding and after wintering. Base group for comparisons is indicated in parentheses. Significant results are highlighted in bold. Abbreviations: HSW: honey-sugar water; ALL THREE: cockroach, cricket and mealworm.

Comparisons	Increase in the # of workers during the treatment				Decrease in the # of workers after wintering			
Carbohydrate diets (HSW)	Estimate	SE	Z	*p*	Estimate	SE	Z	*p*
Honey water	1.16	10.30	0.11	0.910	−0.02	0.23	−0.09	0.929
Sugar pie	−19.27	10.30	−1.87	0.061	0.41	0.23	1.81	0.070
Sugar water	**−36.62**	**10.30**	**−3.55**	**< 0.001**	0.09	0.23	0.39	0.696

Protein diets (ALL THREE)	Estimate	SE	Z	p	Estimate	SE	Z	p
Cockroach	−0.01	0.08	−0.08	0.935	**−0.40**	**0.17**	**−2.38**	**0.018**
Cricket	**0.35**	**0.08**	**4.37**	**< 0.001**	**−0.52**	**0.17**	**−3.09**	**0.002**
Mealworm	−0.08	0.09	−0.98	0.325	−0.06	0.17	−0.39	0.698

**Table 2:** Summary results of the *post hoc* pairwise comparisons (Tukey’s adjustment) based on the corresponding regression models (Table 1). Significant results are highlighted in bold. Abbreviations: HSW: honey-sugar water; ALL THREE: cockroach, cricket and mealworm.

Comparisons	Increase in the # of workers during the treatment				Decrease in the # of workers after wintering			
Carbohydrate diets	Estimate	SE	Ratio	*p*	Estimate	SE	Ratio	*p*
HSW – honey water	−1.16	10.30	−0.11	0.999	0.02	0.23	0.09	1
HSW– sugar pie	19.27	10.30	1.87	0.248	−0.41	0.23	−1.81	0.267
**HSW – sugar water**	**36.62**	**10.30**	**3.55**	**0.003**	−0.09	0.23	−0.39	0.980
Honey water – sugar pie	20.43	10.52	1.94	0.218	−0.43	0.23	−1.86	0.244
**Honey water – sugar water**	**37.78**	**10.52**	**3.59**	**0.003**	−0.11	0.23	−0.47	0.966
Sugar pie – sugar water	17.35	10.52	1.65	0.357	0.32	0.23	1.39	0.503

Protein diets	Estimate	SE	Ratio	p	Estimate	SE	Ratio	p
ALL THREE – cockroach	0.01	0.08	0.08	1	0.40	0.17	2.38	0.082
**ALL THREE** – **cricket**	**−0.35**	**0.08**	**−4.37**	**< 0.001**	**0.52**	**0.17**	**3.09**	**0.011**
ALL THREE – mealworm	0.08	0.09	0.98	0.758	0.06	0.17	0.39	0.980
**Cockroach** – **Cricket**	**−0.36**	**0.08**	**−4.40**	**< 0.001**	0.12	0.17	0.71	0.893
Cockroach – mealworm	0.08	0.09	0.89	0.808	−0.33	0.17	−1.97	0.200
**Cricket** – **mealworm**	**0.44**	**0.08**	**5.28**	**< 0.001**	**−0.45**	**0.17**	**−2.68**	**0.037**

**Fig. 4:**
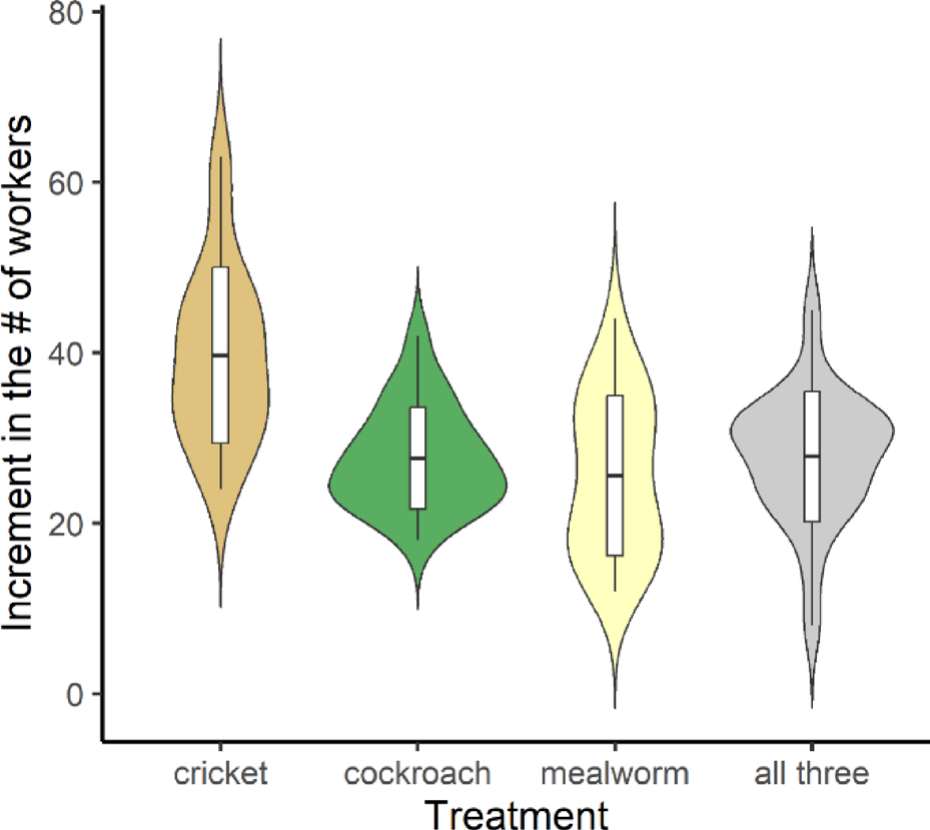
Increment in the number of workers during different protein treatments. Boxes indicate mean and standard deviation values within each group.

**Fig. 5:**
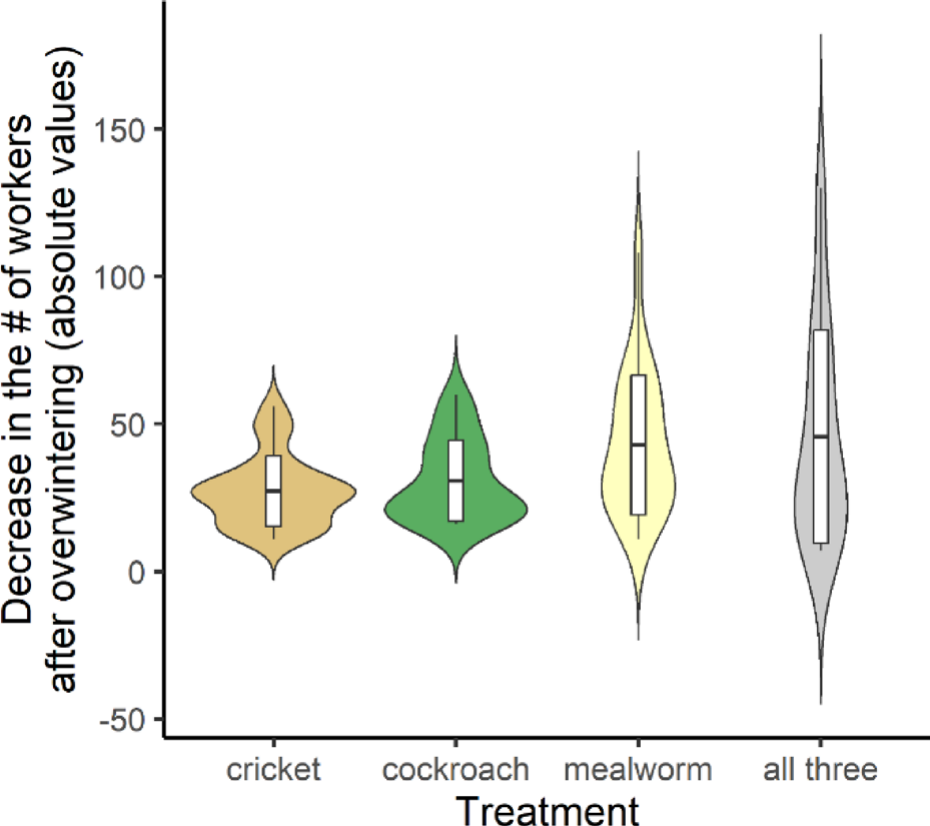
Decrease in the number of workers (absolute values) after the wintering period of colonies on protein diets. Boxes indicate mean and standard deviation values within each group.

In colonies fed different protein diets, the values followed a non-normal distribution, both for increases in the number of workers during the feeding period (W = 0.97, *p* = 0.06) and drops in the number of workers after the wintering phase (W = 0.84, *p* < 0.001). The negative binomial regression models (Table 1) and the *post hoc* comparisons revealed significant differences in the effects of the different protein sources. Colonies that were given crickets had more workers during the treatment period (Fig. 4; Table 2) and showed lower levels of loss after the wintering period than the colonies that were not given crickets (Fig. 5; Table 2).

## Discussion

The influence of four distinct carbohydrate-based diets and four distinct protein-based diets was tested from the perspectives of colony growth and worker survival during wintering for two years. According to our experiments, different diets influence the growth dynamics of ant colonies in different ways: the most effective diets include carbohydrate sources that contain honey and crickets as a protein source.

As proteins are the basic nutritional component of an ant’s body^6^, it may seem obvious that ant colonies should be given protein-rich diets. However, some studies indicate that a protein-rich diet increases mortality among workers^27,28^. Therefore, it is necessary to provide the optimal proportion of carbohydrates and protein to nurture healthy colonies^29–31^.

The most suitable basic artificial diet seems to be a diet with sugar solutions with added amino acids^4,11^. Working with a standardised artificial ant diet for a time could be important in the case of some laboratorial tests, but some elements of a natural diet may also be good for the development of ant colonies^22,25,26,32–35^. These elements would include natural proteins, carbohydrates, vitamins, and minerals. Some articles touch on the potentials of natural diets, but these articles tend to address the uses of these dietary elements for baiting, and the effects of these diets on developing ant colonies are largely overlooked^33–35^.

Our results clearly show that carbohydrate diets that were based on honey can provide better nutrition for ant colonies than diets which only contains sucrose. The best-suited carbohydrate sources were based on honey (Figs. 2 and 3; Tables 1 and 2). Among the four protein diets, colonies that were fed crickets produced exceptionally good growth, while the other three diets produced similar results in the development of the colonies (Figs. 4 and 5; Tables 1 and 2). Similarly, a recent work found that cricket powder performed better than all other protein sources in artificial diets^25^.

Social insects such as ants from the temperate and cold region adapted to seasonal changes, especially during the hibernation period^36,37^. To survive this critical period, the colony has to be prepared for the temperature decrease and the lack of food^38,39^ by a nutritious diet. As our results showed, the type of food that the colonies were given had an influence on the mortality of workers (A.T. and N.Sz., personal observations). These findings were also confirmed by Gutiérrez et al.^29^. Furthermore, conditions during the hibernation period can influence the immune system of ants and their resistance to different forms of contamination^40^. Interestingly, the different carbohydrate diets did not seem to have any effect on mortality among workers during the wintering period (Fig. 3; Tables 1 and 2). This was not true of the different protein diets, since the inclusion of crickets in the diet seemed to have a positive effect on survival (decreased mortality) among workers during the wintering period (Fig. 5; Tables 1 and 2).

Our results confirm that it is worth considering the effects of artificial and natural diets given to ant colonies in laboratory conditions. It is thus essential to arrive at meaningfully comparable results with regards to the effects of diet on ants reared under laboratory conditions. There are many factors which merit testing in the future, e.g. the concentration of sugar and/or honey, the type of honey, other insect species as protein diets and the menu of the insects used as a protein source. It also would be worth doing biochemical analysis on the diets to get information about the background of the effectiveness of the different menus. Finally, we should emphasize that different diets can be suitable in different ways in the case of different ant species, so our results for *Lasius niger* should be interpreted with caution when considering their relevance to other ant species. In other words, different ant species may require different standardised diets. According to these, it would be worth repeating this study with more ant species to see whether a honey water and cricket menu could be a specifically good diet for different species with various dietary characteristics. It should be noted that such a menu could not be suitable for some species feeding on a special diet, like e.g. leafcutter ants.

## Methods

### Model organism

The Formicinae *Lasius* s.str. ants have a Holarctic distribution, and they are among the most common insects in various terrestrial habitats^41–43^. The type species of the *Lasius* genus is *Lasius niger*, and it is the most common ant in many parts of the Eurosiberian region, as the commonly used names for it imply (black garden ant/common black ant)^41–43^. The nuptial period of *L. niger* is summer, when huge numbers of sexuals often fly over a large area at the same time^41–43^, e.g. we easily collected 240 *L. niger* queens on 6 July 2021 within a short time at the campus of the University of Debrecen^22^. This widespread, common species, which is easy to keep and of which numerous colony-founding queens can be easily collected in a short time, makes it easy and efficient to use *L. niger* as a model species of ant. The agricultural relevance of the *L. niger* also makes it a model organism, as it is a common and aggressive aphidicolous and carnivorous ant on plantations, where it cultures aphid colonies and preys on pests in the soil, on the soil surface, in the herb layer, and in tree canopies^18,42–45^.

## Experimental details

To test different diets, 100 young *Lasius niger* colonies^22^ were separated randomly into four groups at the beginning of the experiment (Supplementary Table). The number of workers in each colony was counted and recorded. The colonies were kept in stress-free, dark places in an air-conditioned room at a constant temperature (22.5 ± 0.5 °C) during the experiment. The arena for each colony was a 16.5 cm×11.5 cm×6 cm plastic box in which there was a 12 mm diameter and 100 mm long test tube for nesting. The inner third of the test tube was filled with water and separated by cotton wool. The inner wall of the plastic box was treated with Fluon to prevent the ants from escaping. The top of the box was removed only during the feeding procedure to prevent the ants from escaping. To ensure air ventilation, a 35 mm diameter hole was cut at the top of the box. The hole was covered by a dense metal net (Fig. 1).

During the first experiment (8 April 2022–3 November 2022), four carbohydrate sources were tested: sugar water, honey water, sugar-honey water, and a mixture of powdered sugar and honey (Supplementary Table). To determine the sugar content of the acacia honey to provide the same (40%) sugar concentration for each of the different solutions, a recent study by a Hungarian research group was used^46^. Drawing on the findings of this study, the following solutions were used as carbohydrate sources during the experiment. The sugar water consisted of 40%(m/m) sucrose and 60%(m/m) tap water. The honey water contained 55%(m/m) acacia honey and 45%(m/m) tap water. The honey-sugar water consisted of 20%(m/m) sucrose, 27.5%(m/m) acacia honey, and 52.5%(m/m) tap water. All the solutions were mixed weekly (on Mondays). The fourth diet consisted of a mixture of powdered sugar and acacia honey which was malleable like dough (with a mass ratio of 3:1; “sugar pie” in the followings). From the beginning to 3 August, 20 µl and after 3 August 40 µl (due to the growth of the colonies) the ants were fed with sugar water, honey water, honey-sugar water, or sugar pie (the portion was the size of a head of a pin). These menus were provided for the ants with the same feeding frequency (three times a week, on Mondays, Wednesdays, and Fridays). The standard droplet was always measured with an automatic pipette inserted directly into the “feeders” (the feeder equipment was a LEGO eye for each box), which were replaced with clean pipettes every week. Furthermore, to provide protein for the colonies, each colony received Jamaican field cricket [*Gryllus assimilis* (Fabricius, 1775)] a 3–4 mm in size twice a week (on Mondays and Fridays). The crickets were ordered from the Bugs-World.com, who sent them with some wheat bran, and we gave them a slice of apple. The crickets were fed on this wheat bran and apple menu ad-libitum for a week, then we moved them to a freezer until giving them to the ants. The residue of the cricket was removed at the next feeding time. At the end of the first part of the experiment (3 November 2022), the number of workers was counted and recorded again, and all the colonies were put into refrigerators for wintering. The hibernation period started on 8 November 2022 at 16 °C, and the required temperature (7 °C) was reached by reducing it by 1 °C per day. At the end of the hibernation period (23 March 2023), the temperature was increased by 1 °C per day until it reached 16 °C. After this time (5 April 2023), the workers were recounted to monitor the decrease in their numbers as an effect of wintering.

Since the carbohydrate and protein treatments were independent, the colonies from the first experiment were randomly rearranged into four new groups for the second experiment (Supplementary Table). These groups were given different protein diets (between 5 April 2023 and 17 November 2023) Jamaican field cricket (*Gryllus assimilis*) (1.2–1.6 cm), one Turkestan cockroach [*Blatta lateralis* (Walker, 1868)] (0.8–1.4 cm), two mealworms [*Tenebrio molitor* (Linnaeus, 1758)] (2.0–2.5 cm), and all three alternately. All these animals were ordered from the Bugs-World.com and were fed and stored the same way as the crickets in the previous experiment. So, all of them had the same wheat bran and apple menu ad-libitum for a week before being frozen. The residue of the animals was removed at the next feeding time. During the experiment, 40 µl of honey-sugar water was used as carbohydrate source three times a week (on Mondays, Wednesdays, and Fridays), in addition to the animals (the Jamaican field cricket, the Turkestan cockroach, and the mealworms) which were given to the colonies twice a week (on Mondays and Fridays). At the end of the second part of the experiment (18 November 2023), the number of workers was counted and recorded again, and all of the colonies were put into refrigerators for wintering (from 18 November 2023 to 11 April 2024) according to the procedure described above. At the end of the hibernation period (11 April 2024), the number of workers was counted and recorded.

To eliminate availability of food as a factor that might influence the results, in both experiments the colonies were given more carbohydrates and proteins than they would need to survive. The food remains confirmed that food was available practically ad-libitum for all the colonies.

### Statistical analysis

The increase in the number of workers in each colony during the treatments and the drop in the number of workers after the wintering period were calculated. Data for colonies in which the queen had died were excluded prior to further analysis. First, the normality of the response variables was checked by visual investigation of histograms and by applying Shapiro-Wilk’s test^47^. Levene’s test was also conducted to check homogeneity of variance across groups^48^ for normally distributed variables. Linear regression and generalized linear regression models on normally and negative binomially distributed response variables were performed, respectively. In all cases, treatment was the predictor variable. Then, *post hoc* pairwise comparisons (Tukey’s adjustment) of treatments were applied to evaluate their effects on the number of workers. All statistical analyses were performed in R v4.2.2^49^ including the packages ‘car’^50^ for Levene’s test, ‘glmmTMB’^51^ for regressions and ‘emmeans’^52^ for the *post hoc* comparisons.

## Electronic supplementary material

Below is the link to the electronic supplementary material.

Supplementary Material 1

## Data Availability

All data generated or analysed during this study are included in this published article [and its supplementary information file].
